# Unbiased data mining identifies cell cycle transcripts that predict non-indolent Gleason score 7 prostate cancer

**DOI:** 10.1186/s12894-018-0433-5

**Published:** 2019-01-07

**Authors:** Wendy L. Johnston, Charles N. Catton, Carol J. Swallow

**Affiliations:** 1 Radiation Medicine Program, Princess Margaret Cancer Centre, University Health Network, Toronto, ON Canada; 2 Department of Radiation Oncology, University of Toronto, Toronto, ON Canada; 3 Lunenfeld-Tanenbaum Research Institute, Mount Sinai Hospital, Toronto, ON Canada; 4 Department of Surgery, University of Toronto, Toronto, ON Canada; 5 Institute of Medical Science, University of Toronto, Toronto, ON Canada; 6 Laboratory Medicine and Pathobiology, University of Toronto, Toronto, ON Canada

**Keywords:** Prostate cancer prognosticators, Gleason 3 + 4 = 7 prostate cancer, Biochemical recurrence, Castration-resistant prostate cancer, Biomarker, HES6, E2F2, Cell cycle, BUB1, SRD5A2

## Abstract

**Background:**

Patients with newly diagnosed non-metastatic prostate adenocarcinoma are typically classified as at low, intermediate, or high risk of disease progression using blood prostate-specific antigen concentration, tumour T category, and tumour pathological Gleason score. Classification is used to both predict clinical outcome and to inform initial management. However, significant heterogeneity is observed in outcome, particularly within the intermediate risk group, and there is an urgent need for additional markers to more accurately hone risk prediction. Recently developed web-based visualization and analysis tools have facilitated rapid interrogation of large transcriptome datasets, and querying broadly across multiple large datasets should identify predictors that are widely applicable.

**Methods:**

We used camcAPP, cBioPortal, CRN, and NIH NCI GDC Data Portal to data mine publicly available large prostate cancer datasets. A test set of biomarkers was developed by identifying transcripts that had: 1) altered abundance in prostate cancer, 2) altered expression in patients with Gleason score 7 tumours and biochemical recurrence, 3) correlation of expression with time until biochemical recurrence across three datasets (Cambridge, Stockholm, MSKCC). Transcripts that met these criteria were then examined in a validation dataset (TCGA-PRAD) using univariate and multivariable models to predict biochemical recurrence in patients with Gleason score 7 tumours.

**Results:**

Twenty transcripts met the test criteria, and 12 were validated in TCGA-PRAD Gleason score 7 patients. Ten of these transcripts remained prognostic in Gleason score 3 + 4 = 7, a sub-group of Gleason score 7 patients typically considered at a lower risk for poor outcome and often not targeted for aggressive management. All transcripts positively associated with recurrence encode or regulate mitosis and cell cycle-related proteins. The top performer was BUB1, one of four key MIR145-3P microRNA targets upregulated in hormone-sensitive as well as castration-resistant PCa. SRD5A2 converts testosterone to its more active form and was negatively associated with biochemical recurrence.

**Conclusions:**

Unbiased mining of large patient datasets identified 12 transcripts that independently predicted disease recurrence risk in Gleason score 7 prostate cancer. The mitosis and cell cycle proteins identified are also implicated in progression to castration-resistant prostate cancer, revealing a pivotal role for loss of cell cycle control in the latter.

**Electronic supplementary material:**

The online version of this article (10.1186/s12894-018-0433-5) contains supplementary material, which is available to authorized users.

## Background

In developed countries, prostate cancer (PCa) is the most commonly diagnosed non-skin cancer in men and a leading cause of cancer death [1, 2]. Data compiled in 2010 for Canadian men, for example, showed a 14.1% lifetime risk for developing PCa, and a 3.5% probability of dying from PCa [3]. As such, PCa is second only to lung and bronchus for cancer-related mortality in men.

In PCa patients who have had their prostate removed, biochemical recurrence (BCR) of blood prostate-specific antigen (PSA) occurs in ~ 20–40% of cases within 10–15 years [4–6]. BCR is commonly used as a surrogate for metastatic PCa, since it is a necessary antecedent [7]. However, BCR can occur without radiological evidence of metastasis, and the reported PCa-specific mortality in patients with BCR is only 19–45% by 10–15 years [4, 6, 8]. Prostate tissue is androgen-sensitive, and treatments for metastatic PCa typically include androgen-deprivation. While initially successful, within 2 years ~ 70% of metastatic PCa becomes resistant to androgen deprivation (castration-resistant PCa [CRPC]), leading, almost invariably, to PCa-specific mortality (reviewed in [9]). Given the heterogeneous outcome in patients diagnosed with localized PCa, risk stratification of newly diagnosed patients with non-metastatic disease at presentation is critical to inform clinical management, and treatment options include observation and one or more of radical prostatectomy, radiotherapy, and androgen deprivation therapy [10, 11].

Twenty years ago, D’Amico and colleagues [12] proposed a classification scheme assessing risk for BCR following radical prostatectomy based on PSA, Gleason score and tumour category. Low risk was defined as PSA < 10 ng/ml, Gleason score < 6, and tumour T1-T2a; intermediate risk as PSA 10–20 ng/ml, and/or Gleason score 7, and/or T2b; and high risk as PSA > 20 ng/ml and/or Gleason score 8–10 and/or > T2c. Tumour sub-staging has since been abolished by the AJCC Eighth Edition and by the International Society for Urologic Pathology (https://www.ncbi.nlm.nih.gov/pubmed/27251951). However, tumours can still be stratified within groups; for example, patients with Gleason score 7 tumours are frequently sub-classified into 3 + 4 = 7 (3, primary, 4; secondary, patterns), or 4 + 3 = 7, since 3 + 4 = 7 has a 3-fold lower risk of lethal outcome than 4 + 3 = 7 [13]. Despite these refinements, significant heterogeneity is apparent in the observed incidence of BCR and the more clinically relevant PCa-specific mortality. Heterogeneity is particularly evident for intermediate risk patients, who comprise the largest group [14, 15]. Enhanced discrimination amongst these patients would facilitate more tailored management. As such, new biomarkers are required to accurately identify patients who are at a higher risk for aggressive disease and therefore merit more aggressive treatment, while at the same time allowing for patients with more indolent disease to be observed. Of particular interest are biomolecule markers isolated from tumours, blood, or urine, including proteins, coding and non-coding RNA, and genetic and epigenetic modifications. Unbiased co-discovery of molecules that act in shared cellular pathways might also suggest particularly vulnerable cell signaling pathways to target for therapeutic intervention.

Next generation sequencing with public sharing of data has yielded an enormous amount of genomic and transcriptomic information that is now available to PCa researchers worldwide [16, 17]. An unprecedented and still emerging picture of genome alterations, epigenetic landscape, and gene and protein expression in tumours and normal tissue has enabled the development of a number of PCa prognostic systems (reviewed in [18–20]). Interestingly, there is not much overlap between most previously identified biomarkers [20]. This may reflect, in part, different strategies for biomarker discovery. For example, some studies have queried restricted groups of genes (e.g., a subset of cell cycle genes [21], annotated PCa-associated genes [22], miRNA-regulated transcripts [23]), or different cell types (e.g. stromal cells [24]).

In this study, we exploit recently developed web-based tools and publicly available PCa transcriptome data to perform an unbiased query of multiple datasets, focusing on Gleason score 7 PCa. We identify 12 transcripts that predicted BCR (1 downregulated, 11 upregulated in tumours). Ten of these transcripts remained prognostic for poor outcome in patients with Gleason score 3 + 4 = 7 PCa. Importantly, this suggests a possible strategy to identify higher risk patients within a group that is, overall, considered at a low to intermediate risk for poor outcome. Multivariable logistic regression analysis within the 3 + 4 = 7 group showed combining either UBE2C or CCNB1, which are elevated in PCa, with SRD5A2, which is decreased in PCa, increased the prognostic power over and above any of the 3 transcripts alone. Ten of the 11 Gleason 7 (and all of the Gleason 3 + 4 = 7 sub-group) upregulated biomarkers are mitosis and cell cycle related genes that are also part of an E2F1 transcription-factor regulated cell cycle network that predicts lethal metastatic CRPC [25]. Therefore, in addition to predicting BCR, they are prognostic for PCa-specific mortality. Future studies comparing the performance of these transcripts with other biomarkers in new datasets will help determine their robustness in predicting, and potentially treating, non-indolent PCa.

## Methods

**Fig. 1:**
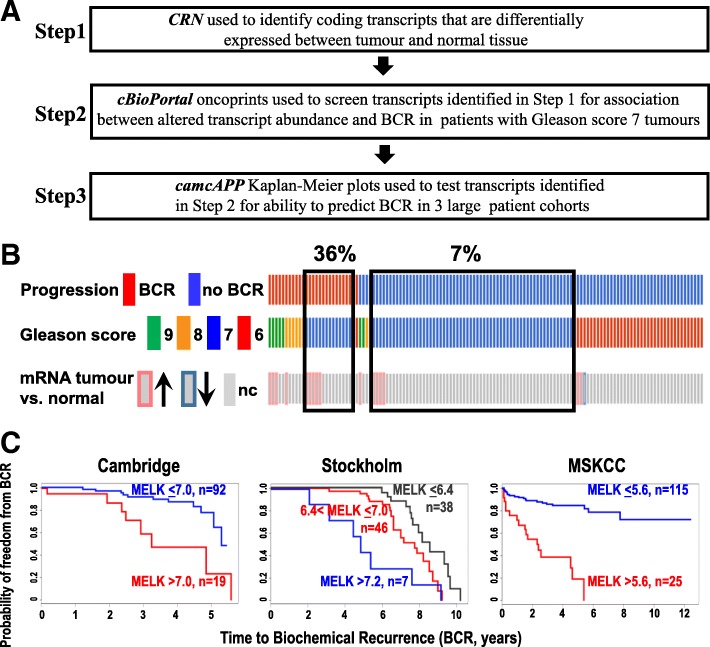
Rapid generation of a transcript test set using 3 web-based tools: CRN, cBioPortal, and camcAPP. **a**) Outline of steps used to generate the transcript test set. **b**) cBioPortal oncoprint (step 2) example (MELK) indicates 36% of patients with Gleason score 7 tumours who experienced biochemical recurrence (BCR) had increased MELK transcript. In contrast, only 7% of those who remained BCR-free had increased transcript. Patients (individual bars) are aligned within the three rows**.** nc**-** no difference in tumour transcript abundance compared with normal tissue. **c**) camcAPP Kaplan-Meier plots (step 3) example (MELK) shows higher MELK expression is associated with shorter time to BCR in all three datasets. Grouping of samples determined by recursive partitioning (Hothorn et al., 2006). **b**, **c**) MSKCC data from Taylor et al., 2010. **c**) Cambridge and Stockholm data from Ross-Adams et al., 2015

A set of test transcripts was identified in an unbiased, stepwise fashion using 3 free, publicly available web-based data visualization and analysis tools that enable rapid genome-wide screening (Fig. 1a). In step 1, Cancer RNA-Seq Nexus (CRN) TCGA-PRAD data (http://syslab4.nchu.edu.tw/) [26] was used to identify genes that had at least one isoform with altered transcript abundance in tumours, compared to normal controls (*p* < 1.0E-04). DAVID 6.8 Gene Name Batch Viewer (https://david.ncifcrf.gov/list.jsp) [27, 28] was used to remove duplicates. In step 2, genes identified in step 1 were queried for patient BCR status, tumour transcript abundance (z-score threshold of 2.0), and tumour Gleason score using cBioPortal, (http://www.cbioportal.org/) [29, 30] MSKCC 2010 data [31]. MSKCC data was used since it is the only cBioPortal prostate cancer dataset in which tumour transcript abundance is compared to normal tissue (other datasets compare patient tumour sample transcript abundance to all tumours that are diploid). In this cohort, 131 patients had primary tumour transcript data available, with 27 of these patients experiencing BCR. Of the 27, 14 had Gleason score 7 tumours and 4 had PSA 10–20 ng/ml. Taking into account the limited number of patients who experienced BCR, we chose to screen transcript abundance in patients with Gleason score 7 tumours. Batches of oncoprints for 50 genes were examined manually to identify transcripts that were increased or decreased in Gleason score 7 patients with BCR, compared to Gleason score 7 patients who remained free of BCR (Fig. 1b shows MELK as an example). In step 3, transcripts identified in Step 2 were then examined using camcAPP (http://bioinformatics.cruk.cam.ac.uk/apps/camcAPP/) [32]. Kaplan-Meier plots examining the relationship between transcript abundance and time to BCR were generated for each transcript using Cambridge, Stockholm, [33] and MSKCC [31] data (Fig. 1c shows MELK as an example). Genes for which the time to BCR could be separated by recursive partitioning [34] into distinct transcript abundance groups for all three datasets were designated the test set.

Expression (transcript per million; TPM) of test genes was examined in TCGA-PRAD normal tissues (*n* = 52), and tumours for which transcript abundance, BCR status, and reviewed Gleason score were available (*n* = 285). Details of sample acquisition and processing following radical prostatectomy at authorized tissue source sites are provided by TCGA-PRAD [35]. Reviewed Gleason score (assigned by TCGA genitourinary pathologists on prostatectomy specimens) was used to avoid possible collection site variability in scoring. Samples with zero or near zero (TPM < 1.6E-07) transcript abundance were removed from the analysis; these included 1 normal for DLGAP5 and CDK1, 3 normals for E2F2 and MELK, and 5 normals and 6 tumours for CDKN3. Univariate logistic regression (SAS 9.4) was performed to determine odds ratios (ORs), and ROC-AUC was calculated for each of the test transcripts, as well as for Gleason score and pre-operative PSA using easyROC http://www.biosoft.hacettepe.edu.tr/easyROC/ [36]. Multivariable logistic regression was performed on transcripts with increased abundance and ORs having *p* values < 0.05, and SRD5A2, which had decreased abundance, in Gleason score 7 (*n* = 158), Gleason score 4 + 3 = 7 (*n* = 70) and Gleason score 3 + 4 = 7 (*n* = 88) tumours. The multivariable model was restricted to include only 2 independent variables due to the limited number (n=13) of BCR events in patients with Gleason score 7 tumours. Pre-operative PSA concentration was only available for a subset of patients (88/156) that included only 4 patients with BCR, so was not included in the multivariable analysis. Future studies that include more samples with longer term follow-up (and hence, more BCR) will be helpful to test the independent contributions of PSA in patients with Gleason score 7 tumours.

Transcripts identified in the current study were examined for overlap with previously reported prognostic transcript signatures [21–23, 25, 33, 37–56]. Gene names were standardized using GeneCards http://www.genecards.org/ v4.5.0 Build 38, except for TSBP, for which 2 aliases were listed in GeneCards (CTR9, c6orf10), and Gene Ontology (GO terms) were determined using DAVID 6.8 https://david.ncifcrf.gov/home.jsp [27, 28]. Proportional Venn diagrams were generated using euler*APE* http://www.eulerdiagrams.org/eulerAPE [57].

## Results

A comparison of prostate cancer primary tumours (*n* = 497) vs. normal prostatic tissue samples (*n* = 52) revealed altered transcript abundance of at least one isoform of 8187 genes (Additional file 1: Table S1). Manual examination of these 8187 genes in MSKCC Gleason score 7 patients using cBioPortal oncoprints identified 1816 genes with differences in expression between patients without BCR (*n* = 60) and those with BCR (*n* = 14) (Additional file 1: Table S2). For each of the 1816 genes identified by the above algorithm, the Cambridge (*n* = 111), Stockholm (*n* = 92), and MSKCC (*n* = 140) datasets were interrogated for any correlation between transcript abundance and BCR-free survival across all Gleason scores. The level of expression of twenty-two individual genes predicted BCR-free survival in all three of the patient cohorts (Table 1). Two of these genes were eliminated from the test set due to discordance in the expression differential between data sets. In particular, for ANKMY1, higher BCR-free survival was observed in association with lower expression in the Cambridge and MSKCC datasets, but with higher expression in the Stockholm dataset. In the case of ALDH1A2, there were discordant results between the MSKCC cohort of Gleason score 7 patients queried using cBioPortal, and the group of patients in the Cambridge, Stockholm, and MSKCC datasets examined using camcAPP. The final test set of 20 transcripts included 13 with higher expression in tumour vs. normal tissue that were positively associated with BCR, and 7 with decreased expression in tumours that were negatively associated with BCR (Table 1).

**Table 1:** Test set of 20 transcripts that predicted time until BCR

			Cambridge (*n* = 111)		Stockholm (*n* = 92)		MSKCC (*n* = 140)	
Transcript*	Chromosomal location	Fold change^c^	*p* value	RP cutoff ^d^	*p* value	RP cutoff ^d^	*p* value	RP cutoff ^d^
TPX2	20q11.21	3.0	4.80E-05	< 6.96	5.40E-02	< 6.45	5.10E-07	< 6.38
BUB1	2q13	2.8	1.10E-02	< 6.26	9.10E-03	< 6.36	9.40E-07	< 6.27
CCNA2	4q27	2.5	1.00E-02	< 6.99	8.70E-03	< 7.16	6.70E-06	< 6.91
E2F2	1p36.12	1.9	2.90E-03	< 7.04	4.10E-02	< 6.62	1.40E-02	< 9.99
UBE2C	20q13.12	3.4	1.80E-03	< 7.52	5.80E-03	< 8.15	2.00E-06	< 6.62
NCAPG	4p15.31	2.7	2.70E-02	< 6.63	4.60E-03	< 6.77	5.50E-07	< 6.02
CDK1	10q21.2	2.1	3.70E-03	< 7.13	2.20E-03	< 7.13	1.40E-04	< 4.95
CDKN3	14q22.2	2.4	1.50E-02	< 6.83	4.50E-03	< 7.01	1.60E-07	< 5.12
DLGAP5	14q22.3	3.4	4.90E-03	< 6.61	1.90E-02	< 6.63	2.90E-06	< 5.45
MELK	9p13.2	3.3	2.40E-02	< 6.97	6.60E-04	< 6.45	3.50E-06	< 5.6
CCNB1	5q13.2	1.8	1.60E-02	< 7.05	3.00E-03	< 6.83	4.30E-04	< 6.98
TMEM206	1q32.3	1.3	2.90E-02	< 7.87	1.30E-02	< 7.22	2.50E-03	< 7.66
SHMT2	12q13.3	1.7	3.20E-02	< 10.4	3.50E-02	< 10.1	1.10E-03	< 8.57
*ANKMY1* ^*a*^	2q37.3	1.03	4.90E-02	< 7.77	2.20E-03	> 7.29	2.70E-03	< 8.11
SRD5A2	2p23.1	− 3.7	9.10E-03	> 7.11	1.70E-03	> 6.53	1.60E-07	> 7.05
CSRP1	1q32.1	−2.9	1.60E-02	> 12	2.30E-02	> 10.9	2.00E-05	> 9.55
NFIB	9p23-p22.3	− 1.1	7.30E-03	> 11.5	1.90E-02	> 11.15	1.80E-04	> 9.23
PGM5	9q21.11	−3.6	5.70E-03	> 8.59	9.30E-03	> 10.8	4.10E-07	> 7.97
CNN1	19p13.2	−3.1	1.40E-02	> 8.99	3.00E-02	> 8.72	2.40E-07	> 8.72
DES	2q35	−3.1	2.40E-02	> 9.53	4.70E-02	> 8.32	2.70E-05	> 8.99
MPDZ	9p23	−1.4	1.10E-02	> 8.22	1.10E-02	> 7.44	3.40E-03	> 8.43
*ALDH1A2* ^*b*^	15q21.3	− 3.2	3.30E-02	> 8.47	2.50E-02	> 7.55	6.40E-07	> 7.19

*The 20 transcripts shown in bold text were altered in tumours compared to normal tissue, and predicted time until BCR in Cambridge (Ross-Adams et al., 2015), Stockholm (Ross-Adams et al., 2015), and MSKCC (Taylor et al., 2010) datasets

^a^*ANKMY1* was excluded since results were discordant between the datasets

^b^*ALDH1A2* was excluded since results were discordant between cBioPortal and camcAPP portals

^c^ total of all isoforms in tumour compared with normal (TCGA-PRAD)

^d^ expression level cutoff for longer time to BCR, determined by recursive partitioning (RP) (Hothorn et al., 2006)

**Fig. 2:**
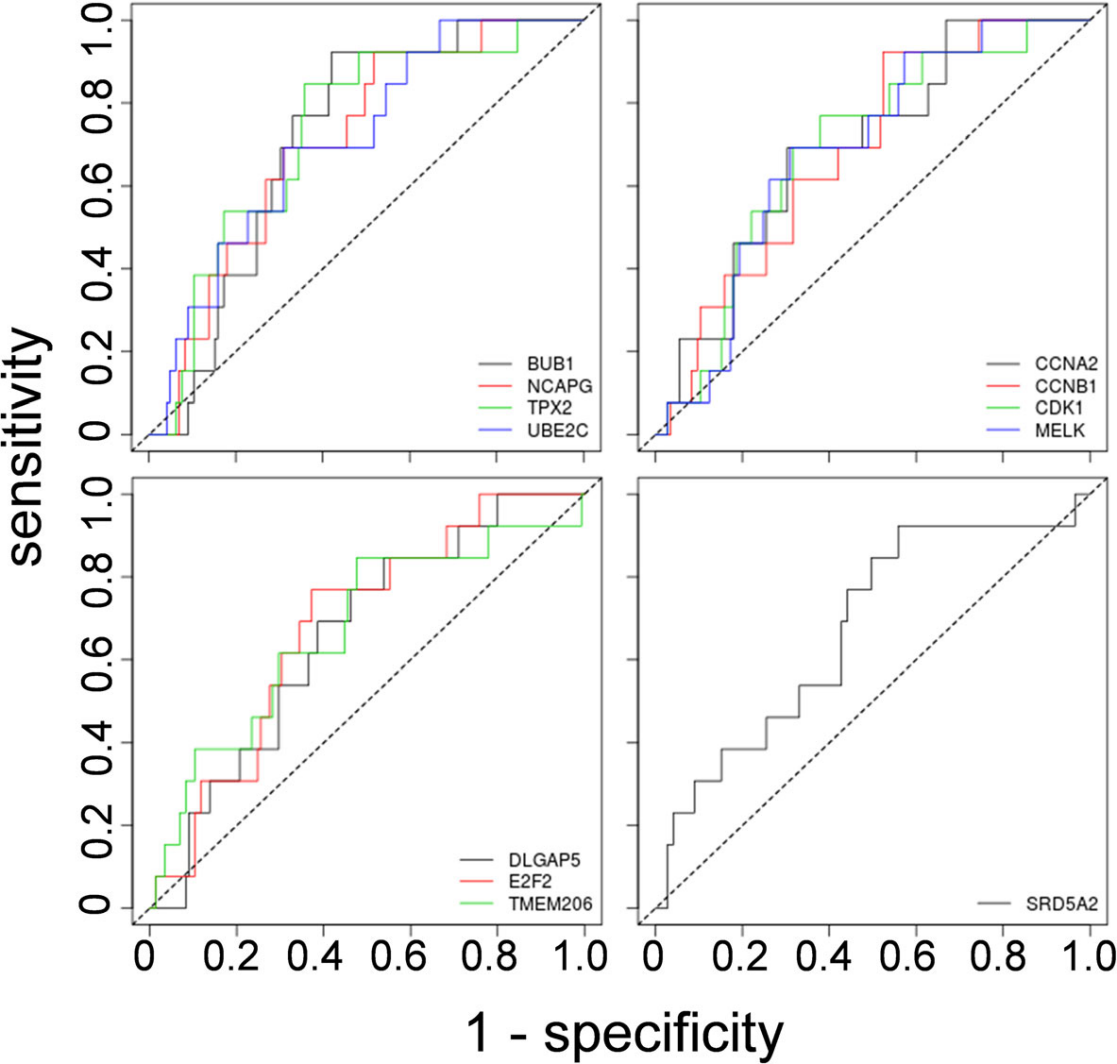
Receiver Operating Characteristic (ROC) curves for transcripts predicting biochemical recurrence (BCR) in patients with Gleason score 7 tumours. (*n* = 158). BCR predicted by increased (BUB1, NCAPG, TPX2, UBE2C, CCNA2, CCNB1, CDK1, MELK, DLGAP5, E2F2, TMEM206), or decreased (SRD5A2) abundance of transcripts. See Table 2 for details. AUC-Area under the curve. Data from TCGA-PRAD (TCGA, 2015)

**Fig. 3:**
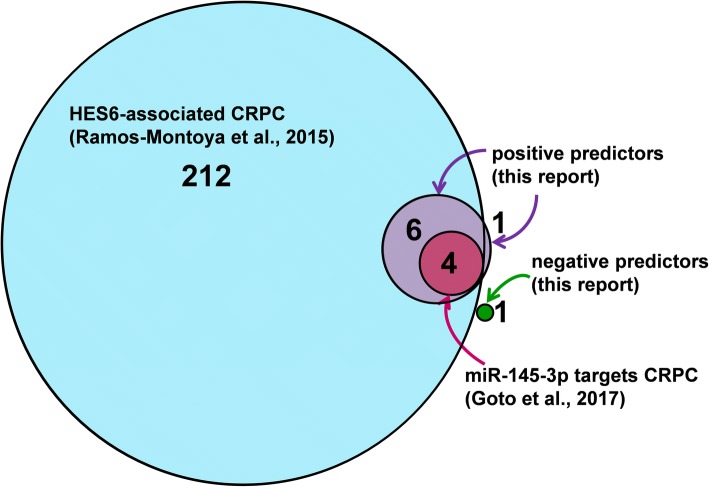
The positive predictors of BCR in Gleason score 7 tumours also predict castration-resistant prostate cancer (CRPC). Ten of 11 positive predictors identified in this study encode cell cycle and mitosis proteins that are part of a HES6-associated E2F1-dependent signature of castration-resistant prostate cancer (CRPC). Four of the 10 are targeted by the miRNA miR-145-3p in CRPC. Total of numbers within or adjacent to each circle are the number of prognostic transcripts in each dataset

When examined across Gleason scores 6 through 10, 14 of 20 test set genes yielded an OR, and 17 an ROC-AUC, that was prognostic (*p* < .05) for BCR in the TCGA-PRAD validation dataset (*n* = 285) (Additional file 1: Table S3). For half of the genes, the AUC was greater than for Gleason score or pre-operative PSA, examined in the same group of patients (Additional file 2). Within the group of Gleason score 7 patients in the TCGA-PRAD dataset (*n* = 158), 12 transcripts remained prognostic (Table 2, Fig. 2), with 1 negatively associated with BCR (SRD5A2) and 11 positively associated with BCR (BUB1, TPX2, NCAPG, UBE2C, MELK, CCNA2, CCNB1,CDK1, E2F2, DLGAP5, TMEM206,). Ten of the 11 transcripts positively associated with BCR encode proteins that are functionally annotated as related to mitotic and cell cycle (GO:0000082, GO:0051726), cell division (GO:0051301), or cell proliferation (GO:0008283). The proteins they encode also participate in an E2F1-dependent cell cycle network associated with CRPC, and 4 of the 11 (BUB1, NCAPG, CDK1, MELK) are implicated in miRNA (MIR145-3P)-dependent CRPC (Fig. 3). The 11th transcript encodes a membrane protein (TMEM206) that has recently been identified as important to control cell proliferation in colorectal cancer cells [58].

**Table 2:** Univariate logistic regression and ROC-AUC analyses for predicting BCR in patients with Gleason score 7 tumours

Variable	Odds Ratio (OR)	95% CI OR	*P* value OR	ROC-AUC	SE AUC	*P* value AUC	Youden cut-off	Sensitivity	Specificity	PPV	NPV
*(up in tumour)*											
BUB1	1.31	0.908–1.889	1.48E-01	0.720	0.055	5.35E-05	1.22	0.92	0.58	0.16	0.99
TPX2	1.18	0.994–1.406	5.79E-02	0.732	0.066	4.53E-04	3.54	0.85	0.64	0.18	0.98
NCAPG	1.51	0.913–2.510	1.08E-01	0.711	0.065	1.08E-03	0.94	0.92	0.48	0.14	0.99
UBE2C	1.07	0.998–1.142	5.60E-02	0.713	0.067	1.42E-03	7.79	0.69	0.69	0.17	0.96
MELK	1.21	0.908–1.612	1.93E-01	0.687	0.065	3.97E-03	1.82	0.69	0.69	0.17	0.96
CCNA2	1.27	0.967–1.680	8.57E-02	0.694	0.069	5.04E-03	2.58	0.69	0.70	0.17	0.96
CCNB1	1.13	0.954–1.345	1.56E-01	0.685	0.066	5.20E-03	4.60	0.92	0.48	0.14	0.99
CDK1	1.07	0.969–1.181	1.81E-01	0.691	0.070	6.27E-03	4.97	0.77	0.62	0.15	0.97
E2F2	2.84	0.715–11.309	1.38E-01	0.682	0.068	7.55E-03	0.37	0.77	0.63	0.16	0.97
DLGAP5	1.24	0.766–2.014	3.80E-01	0.657	0.070	2.54E-02	0.65	0.85	0.46	0.12	0.97
TMEM206	1.15	0.999–1.321	5.10E-02	0.672	0.086	4.51E-02	9.11	0.85	0.52	0.14	0.97
CDKN3	1.14	0.936–1.387	1.93E-01	0.643	0.079	6.93E-02	2.04	0.92	0.39	0.12	0.98
SHMT2	1.01	0.990–1.038	2.52E-01	0.605	0.069	1.25E-01	56.64	0.69	0.57	0.13	0.95
*(down in tumour)*											
SRD5A2	0.90	0.786–1.021	9.85E-02	0.674	0.079	2.70E-02	7.98	0.92	0.44	0.13	0.99
CSRP1	0.998	0.996–1.001	1.30E-01	0.628	0.080	1.11E-01	417.97	0.54	0.72	0.15	0.95
PGM5	0.99	0.974–1.006	2.04E-01	0.592	0.085	2.84E-01	22.95	0.39	0.81	0.15	0.94
NFIB	0.98	0.941–1.029	4.68E-01	0.584	0.092	3.60E-01	27.09	0.69	0.56	0.12	0.95

Data are from TCGA-PRAD (TCGA, 2015). *n* = 158. CI-confidence interval, SE-standard error. Youden cut-off is the optimal point to separate biochemical recurrence (BCR) from non-BCR. PPV-positive predictive value, NPV-negative predictive value

Ten of 12 transcripts that were prognostic in Gleason score 7 tumours remained prognostic in patients with Gleason 3 + 4 = 7 tumours (Table 3), and multivariable logistic regression analysis showed that combining CCNB1 or UBE2C with SRD5A2 slightly improved the predictive power of the model (Additional file 1: Table S4). In low risk (Gleason score 6) tumours, 9 transcripts remained prognostic, although the limited number of cases of BCR (n=3) in this group may limit its predictive power (Additional file 1: Table S5). Interestingly, in Gleason 4 + 3 = 7, only TMEM206 was prognostic (Additional file 1: Table S6). The reason for the discrepancy between results for Gleason 3 + 4 = 7 and 4 + 3 = 7 is unclear. One possibility is that, with the exception of MELK, transcript abundance of positive prognosticators was higher in Gleason 3 + 4 = 7 than in Gleason 4 + 3 = 7 in patients with BCR (Additional file 1: Table S7). Future studies examining transcript abundance within regions of different pattern within the tumour may be helpful to explain these findings.

**Table 3:** Univariate logistic regression and ROC-AUC analyses for predicting BCR in patients with Gleason score 3 + 4 = 7 tumours

Variable	Odds Ratio (OR)	95% CI OR	*P* value OR	ROC-AUC	SE AUC	*P* value AUC	Youden cut-off	Sensitivity	Specificity	PPV	NPV
*(up in tumour)*											
BUB1	1.93	1.029–3.604	4.03E-02	0.865	0.045	4.75E-16	1.54	1.000	0.78	0.22	1.00
TPX2	1.40	1.055–1.852	1.98E-02	0.872	0.060	5.07E-10	4.09	1.000	0.74	0.19	1.00
NCAPG	2.72	1.071–6.915	3.55E-02	0.855	0.067	1.23E-07	1.33	0.800	0.83	0.22	0.99
UBE2C	1.20	1.051–1.369	7.00E-03	0.889	0.050	7.21E-15	7.79	1.000	0.80	0.23	1.00
MELK	1.72	0.939–3.152	7.89E-02	0.819	0.082	1.03E-04	2.17	0.800	0.86	0.25	0.99
CCNA2	1.83	1.113–3.000	1.71E-02	0.810	0.106	3.39E-03	2.63	0.800	0.80	0.19	0.99
CCNB1	1.63	1.109–2.383	1.27E-02	0.824	0.083	1.00E-04	4.69	1.000	0.57	0.12	1.00
CDK1	1.28	1.070–1.532	6.80E-03	0.863	0.090	5.50E-05	7.32	0.800	0.90	0.33	0.99
E2F2	5.98	0.824–43.408	7.70E-02	0.720	0.119	6.39E-02	0.37	0.800	0.70	0.14	0.98
DLGAP5	1.95	0.954–3.984	6.73E-02	0.855	0.066	6.61E-08	0.86	1.000	0.64	0.14	1.00
TMEM206	0.98	0.766–1.243	8.40E-01	0.545	0.154	7.72E-01	9.11	0.800	0.52	0.09	0.98
CDKN3	1.50	1.071–2.108	1.85E-02	0.708	0.187	2.65E-01	6.47	0.600	0.95	0.43	0.98
SHMT2	1.03	0.983–1.075	2.21E-01	0.740	0.093	9.94E-03	60.61	0.800	0.71	0.14	0.98
*(down in tumour)*											
SRD5A2	0.58	0.367–0.921	2.09E-02	0.884	0.079	1.03E-06	3.75	0.800	0.88	0.29	0.99
CSRP1	1.00	0.992–1.001	9.71E-02	0.752	0.132	5.66E-02	417.97	0.800	0.80	0.19	0.99
PGM5	0.97	0.930–1.007	1.04E-01	0.745	0.136	7.28E-02	18.57	0.600	0.90	0.27	0.97
NFIB	0.98	0.903–1.054	5.26E-01	0.578	0.129	5.45E-01	24.07	0.600	0.72	0.12	0.97

Data are from TCGA-PRAD (TCGA, 2015). *n* = 88. CI-confidence interval, SE-standard error. Youden cut-off is the optimal point to separate biochemical recurrence (BCR) from non-BCR. PPV-positive predictive value, NPV-negative predictive value

## Discussion

PCa is a common disease with a heterogeneous clinical outcome that is difficult to predict using available risk stratification tools. Treatments such as prostatectomy, radiotherapy and androgen deprivation extend lifespan and improve the quality of life in patients with aggressive disease, but also cause unnecessary morbidity and loss of quality of life in those with indolent disease. Thus, a key goal in disease management is to develop biomarkers that accurately predict outcome. Variable outcome is especially evident in “intermediate risk” patients, making clinical decision-making particularly difficult within this group. To identify biomarkers that may be helpful for predicting outcome in this group, we took the approach of broadly querying publicly available transcriptome data using multiple distinct visualization and analysis data portals. We identify 12 genes, the expression of which predicted poor outcome (BCR) in patients with Gleason score 7 tumours. Eleven of the twelve genes have been identified as prognostic for PCa outcome in at least one other study (Additional file 1: Table S8), portending robustness. Importantly, 10/12 of the genes remained prognostic in Gleason score 3 + 4 = 7 tumours. Since Gleason 3 + 4 = 7 tumours are often projected to have a more favourable prognosis [59], the identification of transcripts that may stratify this group into higher and lower risk of poor outcome may be particularly helpful for clinical decision-making.

The top performer we identified in Gleason score 7 tumours, including Gleason 3 + 4 = 7, was BUB1. BUB1 encodes a mitotic checkpoint Ser/Thr kinase that has recently been implicated as a key regulator of prostate cancer progression [60]. TPX2 was also a top prognosticator and is a microtubule-associated protein that stimulates Ran-GTP-dependent microtubule nucleation and regulates Aurora A kinase during mitosis and cell cycle progression [61, 62]. Elevated expression of TPX2 is a common finding in human cancers, including prostate, and overexpression in vitro increases invasion of multiple cancer cell lines [63–65]. Like BUB1 and TPX2, the other transcripts positively associated with BCR function in the cell cycle and/or mitosis. Elevated abundance of cell cycle and mitosis transcripts has previously been shown to predict PCa outcome, and is the basis of the Prolaris commercial test for predicting PCa aggressiveness [21].

The cell cycle and mitosis genes identified here are associated with HES6-dependent E2F1 transcription factor-mediated CRPC [25]. HES6 is a transcription cofactor that physically interacts with E2F1 as well as the androgen receptor [25]. During the G1/S transition, cyclin-dependent kinases and cyclins phosphorylate the tumour suppressor retinoblastoma, resulting in a weakening of its interaction with E2F proteins [66]. E2F2 (as well as E2F1 and E2F3a) are then free to activate genes that promote S phase entry and cell cycle progression. In CRPC, HES6 is able to maintain androgen receptor activity in the absence of testosterone. In the current study E2F2, but not E2F1, was found to be prognostic for BCR, indicating E2F2 may be a more sensitive or earlier indicator of poor clinical outcome than the related family member, E2F1.

E2F2 transcription factor has been shown previously to be negatively regulated by the Let-7 family miRNA, MIRLET7A, leading to suppression of growth of PCa cells [67]. miRNAs are small RNA molecules that are bound by Argonaute proteins to form an RNA-induced silencing complex that targets specific mRNA(s), typically leading to translational repression [68]. The abundance of many miRNA is altered in PCa, and is prognostic for clinical outcome [69]. Some miRNA, such as MIRLET7A, act as tumour suppressors by targeting genes (e.g. E2F2), that enhance tumour growth. In contrast, others act as oncogenes by targeting tumour suppressors or cell death and differentiation pathway mRNAs [70]. MIR145-3P, a tumour suppressive miRNA, is decreased modestly in hormone-sensitive PCa, and decreased severely in CRPC, and low levels are associated with a shorter time to BCR [71]. Examining MIR145-3P in detail, Goto et al. identified four key MIR145-3P targets (BUB1, NCAPG, CDK1, MELK) [23]. All four are upregulated in hormone-sensitive PCa, and elevated even more in CRPC, and all are part of the group of cell cycle and mitosis genes identified in the current study, both in Gleason score 7, and in the Gleason score 3 + 4 = 7 subgroup. Given that thousands of proteins participate in the cell division cycle [72], the specific identification of BUB1, NCAPG, CDK1, and MELK as predictors of BCR, as well as key targets of MIR145-3P that are associated with CRPC, suggests a particularly important role for these cell cycle/mitosis genes in aggressive PCa.

Like other cancers, PCa frequently exhibits defects in cell cycle regulation and cell cycle progression and cell cycle proteins have been explored as therapeutic targets [73]. For example, prostate cancer Phase IB/II trials are ongoing for the CDK4/6 inhibitors ribociclib (ClinicalTrials.gov identifier: NCT02555189, NCT02494921) and palbociclib (NCT02905318, NCT02059213), and the CHEK1 inhibitor LY2606368 (NCT02203513). The cell cycle and mitosis proteins we identified in the present study do not include CDK4/6 or CHEK1. However, almost half are targets of MIRLET7A or MIR145-3P tumour suppressor miRNA. Pre-clinical and clinical trials are currently underway to determine the feasibility of using synthetic miRNA mimics (to compensate for decreased abundance of endogenous tumour suppressive miRNA) or antimiRNA (to block endogenous oncogenic miRNA) as therapeutic agents [70]. Diseases currently being targeted in clinical trials include hepatitis (NCT02508090, NCT02452814), lymphoma (NCT0250552) and mesothelioma (NCT02369198). The experimental evidence supports an important role in PCa progression of miRNA regulation of E2F2, BUB1, NCAPG, CDK1, and MELK. As such, in future studies it would be interesting to determine if the cell cycle and mitosis transcripts identified in the current study might prove to be effective targets for miRNA-based therapeutics in PCa patients.

SRD5A2 was decreased in tumours and negatively associated with BCR. SRD5A2 encodes a steroid 5-α-reductase 2 that converts testosterone to the more potent androgen receptor agonist, dihydrotestosterone. Multivariable logistic regression analysis showed that including CCNB1 or UBE2C and SRD5A2 as variables improved the predictive power in Gleason 3 + 4 = 7. CCNB1 encodes a cyclin B1 required during mitosis, and UBE2C encodes an E2 ubiquitin-conjugating enzyme important in cyclin destruction. These findings suggest it may be beneficial to pair predictors from distinct cellular pathways (cell cycle/mitosis plus androgen synthesis) to improve prognostication in Gleason score 3 + 4 = 7 PCa. SRD5A2 was previously identified in a candidate screen of 732 PCa-related genes [22] and forms part of the commercial Oncotype DX Genomic Prostate Score assay that predicts tumour aggressiveness in low and intermediate risk PCa. SRD5A2 was also identified by Rubicz et al. [50] in an unbiased screen for transcripts that improved prediction of PCa recurrence following radical prostatectomy when combined with Gleason score. Taken together, these findings suggest SRD5A2 may be a robust prognosticator of disease outcome in PCa. However, lower SRD5A2 transcript abundance has previously been reported to be associated with poor [50] or favourable [22] outcome. Moreover, altered SRD5A2 activity resulting from genetic polymorphisms or treatment with drugs such as finasteride has inconsistent effects on PCa outcome [74–76]. Accordingly, additional studies are warranted to clarify the role of SRD5A2 abundance in PCa outcome.

The average patient follow-up time in the TCGA validation dataset was short (< 2 years) [35], consistent with the transcripts identified in the current study as prognostic for early, aggressive disease progression. The small number of patients with BCR in this short follow-up time (13/158 Gleason score 7 cases), together with incomplete data for pre-operative PSA, precluded our testing for concurrent, independent roles for transcript abundance and PSA in the multivariable logistic regression analysis. In the future, prospective studies that include a larger number of patients with complete clinical information and long-term follow-up (15 years+) would be helpful to determine if the improvement in risk stratification we identified here is durable and reproducible.

## Conclusions

In this study we identify SRD5A2, and 11 mitosis and cell cycle transcripts that predict PCa disease progression in patients with Gleason score 7 disease. The prognostic power of 10 of these transcripts extended to patients with Gleason 3 + 4 = 7 disease indicating they are excellent candidates for stratifying risk in this group of patients with disease that is often deemed likely to have a favourable outcome. Future studies, especially those including long-term clinical follow-up, will be helpful to confirm the robustness of their predictive power, as well as the therapeutic potential for pharmacologic or genetic intervention.

## Additional files

Additional file 1: An excel file containing 8 spreadsheets: **Table S1.** (transcripts identified in Step 1 using CRN), **Table S2**. (transcripts identified in Step 2 using cBioPortal), **Table S3.** (univariate logistic regression and ROC-AUC analyses in patients with Gleason score 6–10 tumours), **Table S4.** (multivariable logistic regression analysis for predicting BCR in patients with Gleason score 3 + 4 = 7 using pairs of one upregulated and one downregulated transcript), **Table S5.** (univariate logistic regression and ROC-AUC analyses in patients with Gleason score 6 tumours), **Table S6.** (univariate logistic regression and ROC-AUC analyses in patients with Gleason score 4 + 3 = 7 tumours), **Table S7.** (mean transcript abundance in patients with BCR and without BCR, broken down by Gleason score), and **Table S8.** (description of the 12 transcripts that predicted BCR in the validation dataset. Includes citations for other studies that found these transcripts to be prognostic for PCa outcome). (XLSX 189 kb)

Additional file 2: **Figure S1.** showing ROC-AUC for test transcripts in patients with Gleason score 6–10 tumours. (JPG 1535 kb)

## Abbreviations

BCR: Biochemical recurrence
CRPC: Castration-resistant prostate cancer
miRNA: microRNA
OR: Odds ratio
PCa: Prostate cancer
PSA: Prostate-specific antigen
ROC-AUC: Receiver operating characteristic area under the curve
TPM: Transcripts per million
