# Supplementary figures and images for: Unbiased data mining identifies cell cycle transcripts that predict non-indolent Gleason score 7 prostate cancer
Source: BMC Urol. 2019 Jan 7;19:4. doi: 10.1186/s12894-018-0433-5 (PMC6322345; doi:10.1186/s12894-018-0433-5)

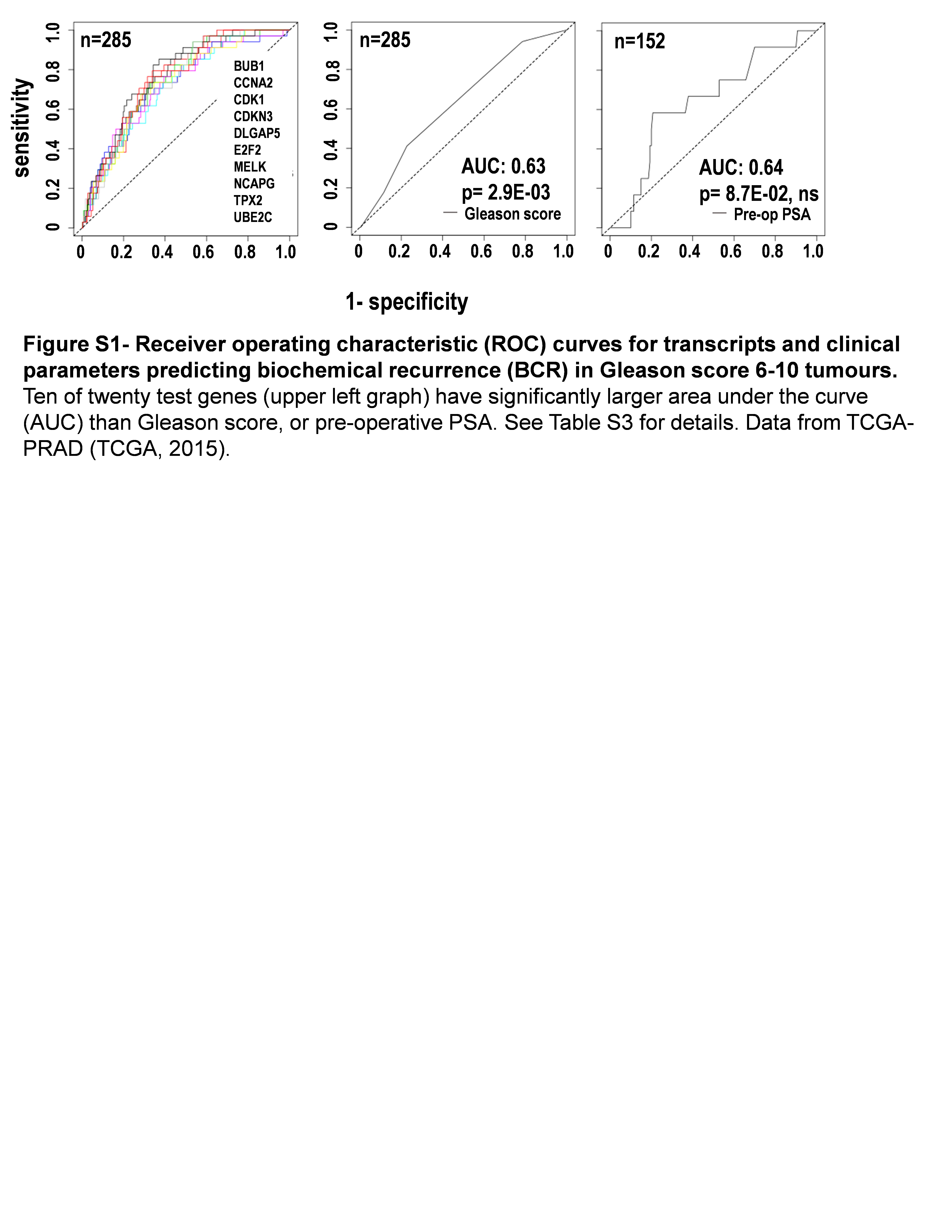

Supplement: Supplementary file 2 — Figure S1. showing ROC-AUC for test transcripts in patients with Gleason score 6–10 tumours. (JPG 1535 kb) [file 12894_2018_433_MOESM2_ESM.jpg]
